# NRXN1α^+/-^ is associated with increased excitability in ASD iPSC-derived neurons

**DOI:** 10.1186/s12868-021-00661-0

**Published:** 2021-09-15

**Authors:** Sahar Avazzadeh, Leo R. Quinlan, Jamie Reilly, Katya McDonagh, Amirhossein Jalali, Yanqin Wang, Veronica McInerney, Janusz Krawczyk, Yicheng Ding, Jacqueline Fitzgerald, Matthew O’Sullivan, Eva B. Forman, Sally A. Lynch, Sean Ennis, Niamh Feerick, Richard Reilly, Weidong Li, Xu Shen, Guangming Yang, Yin Lu, Hilde Peeters, Peter Dockery, Timothy O’Brien, Sanbing Shen, Louise Gallagher

**Affiliations:** 1 School of Medicine, Regenerative Medicine Institute, Biomedical Science Building BMS-1021, National University of Ireland Galway, Dangan, Ireland; 2 Physiology and Cellular Physiology Research Laboratory, School of Medicine, CÚRAM SFI Centre for Research in Medical Devices, National University of Ireland (NUI), Galway, Ireland; 3 School of Mathematical Sciences, University College Cork, Cork, Ireland; 4 Department of Physiology, College of Life Science, Hebei Normal University, Shijiazhuang, China; 5 HRB Clinical Research Facility, National University of Ireland (NUI), Galway, Ireland; 6 Department of Haematology, Galway University Hospital, Galway, Ireland; 7 Trinity Institute of Neuroscience, Trinity College Dublin, Dublin, Ireland; 8 Children’s University Hospital, Temple Street, Dublin, Ireland; 9 Department of Clinical Genetics, OLCHC, Dublin 12, Ireland; 10 School of Medicine and Medical Science, UCD Academic Centre On Rare Diseases, University College Dublin, Dublin, Ireland; 11 Centre for Bioengineering, Trinity College Institute of Neuroscience, School of Medicine, School of Engineering, Trinity College Dublin, Dublin, Ireland; 12 Bio-X Institutes, Key Laboratory for the Genetics of Development and Neuropsychiatric Disorders (Ministry of Education), Shanghai Key Laboratory of Psychotic Disorders, and Brain Science and Technology Research Center, Shanghai Jiao Tong University, Shanghai, China; 13 School of Medicine and Life Sciences, Nanjing University of Chinese Medicine, 138 Xianlin Road, Nanjing, 210023 China; 14 College of Pharmacy, Nanjing University of Chinese Medicine, Nanjing, Jiangsu China; 15 College of Pharmacy, Jiangsu Key Laboratory for Pharmacology and Safety Evaluation of Chinese Materia Medica, Jiangsu Collaborative Innovation Center of Traditional Chinese Medicine (TCM) Prevention and Treatment of Tumor, Nanjing University of Chinese Medicine, Nanjing, 210023 Jiangsu China; 16 Centre for Human Genetics, University Hospital Leuven, KU Leuven, 3000 Leuven, Belgium; 17 Centre for Microscopy and Imaging, Anatomy, School of Medicine, National University of Ireland (NUI), Galway, Ireland; 18 FutureNeuro Research Centre, Royal College of Surgeons in Ireland, Dublin, D02 Ireland

**Keywords:** ASD, Excitability, Induced pluripotent stem cell, Neurexin, RNA sequencing

## Abstract

**Background:**

*NRXN1* deletions are identified as one of major rare risk factors for autism spectrum disorder (ASD) and other neurodevelopmental disorders. ASD has 30% co-morbidity with epilepsy, and the latter is associated with excessive neuronal firing. *NRXN1* encodes hundreds of presynaptic neuro-adhesion proteins categorized as NRXN1α/β/γ. Previous studies on cultured cells show that the short NRXN1β primarily exerts excitation effect, whereas the long NRXN1α which is more commonly deleted in patients involves in both excitation and inhibition. However, patient-derived models are essential for understanding functional consequences of *NRXN1*α deletions in human neurons. We recently derived induced pluripotent stem cells (iPSCs) from five controls and three ASD patients carrying *NRXN1*α^+/-^ and showed increased calcium transients in patient neurons.

**Methods:**

In this study we investigated the electrophysiological properties of iPSC-derived cortical neurons in control and ASD patients carrying *NRXN1*α^+/-^ using patch clamping. Whole genome RNA sequencing was carried out to further understand the potential underlying molecular mechanism.

**Results:**

*NRXN1α*^﻿﻿+﻿﻿/﻿﻿-^ cortical neurons were shown to display larger sodium currents, higher AP amplitude and accelerated depolarization time. RNASeq analyses revealed transcriptomic changes with significant upregulation glutamatergic synapse and ion channels/transporter activity including voltage-gated potassium channels (*GRIN1*, *GRIN3B*, *SLC17A6*, *CACNG3*, *CACNA1A*, *SHANK1*), which are likely to couple with the increased excitability in *NRXN1α*^﻿﻿+﻿﻿/﻿﻿-^ cortical neurons.

**Conclusions:**

Together with recent evidence of increased calcium transients, our results showed that human *NRXN1α*^﻿﻿+﻿﻿/﻿﻿-^ isoform deletions altered neuronal excitability and non-synaptic function, and *NRXN1α*^﻿﻿+﻿﻿/﻿﻿-^ patient iPSCs may be used as an ASD model for therapeutic development with calcium transients and excitability as readouts.

**Supplementary Information:**

The online version contains supplementary material available at 10.1186/s12868-021-00661-0.

## Background

Autism spectrum disorder (ASD) is a lifelong neurodevelopmental disease characterized by social interaction impairment, communication deficits and repetitive behaviors. Clinically, ASD is frequently comorbid with epilepsy, intellectual disability, language delay, severe hyperactivity [1] and premature mortality [2]. Genetically, heterozygous deletion of *NRXN1* is commonly identified as a major rare risk among ASD [3–8], epilepsy [7, 9–12], mental retardation [13], ADHD [14] and schizophrenia [15–18].

NRXN signaling is complex. Human *NRXN1* encodes numerous presynaptic Neurexins by 24 exons over 1.1 Mb genomic DNA, with 3.5 Mb intergenic regions which are highly conserved during evolution. Neurexin proteins were categorized into the long NRXN1α encoded by exons 1–24 and short NRXN1β by exons 18–24. Recently, a third promoter was described to transcribe the shortest Nrxn1γ lacking all extracellular EGF repeats and LNS domains [19, 20]. Whereas the function of NRXN1γ is largely unknown, the NRXN1β is thought to exert primarily excitation effect, and the NRXN1α involves in both excitation and inhibition. They differentially bind to post-synaptic Neuroligins and play key roles in localizing a network of postsynaptic proteins and soluble adaptors to the synapse [21]. Furthermore, three *NRXN1-3* genes are identified to generate thousands of splicing variants [22]. Neurexin proteins were found to form discrete nanoclusters at the excitatory synapse containing > 4 Neurexin-1 molecules together with Neurexin-2 and/or Neurexin-3 isoforms, [23], thus dramatically increasing the complexity and importance of NRXN signaling.

However, *Nrxn1a*^*−/−*^ mice were initially found to display only subtle behavioral deficits in nest building, otherwise viable, fertile and indistinguishable from wildtype littermates [24]. Further studies revealed discrete impairments in synaptic transmission, with a reduced mEPSC frequency in *Nrxn1α*^*−/−*^ CA1 neurons detected by electrophysiological recording [25]. However, there was neither an alteration in neurotransmitter release from a report a decade ago [25], nor a significant phenotype detected in cultured *Nrxn1a*^+/-^ or *Nrxn1a*^*−/−*^ mouse cortical neurons from a recent study [26]. This raises a question if the *Nrxn1a*^*−/−*^ mouse can adequately recapitulate human *NRXN1*^+/-^ phenotypes.

In contrast, human H1 embryonic stem cells (ESC) were engineered to create *NRXN1*^+/-^ by deleting exon 19 or 24, and the derived mutant cortical neurons showed severe impairments in neurotransmitter release [26]. It shall be noted that based on the exon usage, exon 19 deletion lines are *NRXN1a*^+/-^/*NRXN1β*^+/-^ whereas exon 24 truncation lines are *NRXN1a*^+/-^/*NRXN1β*^+/-^/*NRXN1γ*^+/-^. They may be applicable to a minority of patients with deletions at 3′ of the *NRXN1* gene. However, most patients harbor heterozygous deletions in the 5′ region of *NRXN1* and therefore impact the *NRXN1α*^+/-^ isoforms only. In addition, the mutation was created on one genomic background of H1 ESCs (assumingly healthy), and consequently, the model is unlikely to address variable expressivity and pleiotropy of clinical outcomes which may be associated with additional rare/common variants in patients’ genome. Patient-derived cell models are crucial to address as yet unexplained phenomena of variable expressivity and pleiotropy associated with *NRXN1* deletions [8, 27, 28].

A recent study using iPSCs derived from psychiatric patients carrying a deletion at 5′ or 3′ of *NRXN1* gene showed that patient derived cells express dozens of novel isoforms from the deletion allele, in addition to > twofold reduction of long *NRXN1α* isoforms. Neuronal activity in mutant neurons is ameliorated by overexpression of wildtype isoforms, whereas novel mutant isoforms decreased control neuronal activity [29]. This data highlights the complexity of NRXN1 splicing, and underpins the importance of human-derived studies, as not only haploinsufficiency of the wildtype NRXN1 isoforms, but also gain-of-function of mutant isoforms may contribute to pathogenesis and to the observed clinical diversity.

Previously we reported on generating iPSCs from three ASD patients with *NRXN1α*^+/-^ deletions, and showed that ASD *NRXN1α*^+/-^ display increased calcium signalling activity that was sensitive to voltage gated sodium (TTX) and voltage gated calcium (nifedipine,agatoxin) channel blockers [30]. These data were supported by whole genome RNASeq analysis. In this current study, we explore the electrophysiological basis underlying the calcium signalling activity of *NRXN1α*^+/-^ in cultured cortical neurons, derived from iPSCs in three ASD patients compared to five control lines. In addition, a detailed quantitative analysis of the original whole genome RNASeq dataset is stringently applied, to compare transcriptomes between control and patient lines to reveal differentially expressed genes (DEGs) and relevant pathways. Consistent with the ASD hypothesis of excitatory/inhibitory (E/I) imbalance [31][32, 33], we find specific defects in non-synaptic properties of neurons, with significantly increased excitability, upregulated voltage-gated ion channels and glutamatergic signaling, which aligns well with our previously identified phenotype of increased calcium transients in *NRXN1α*^+/-^ neurons [30].

## Results

### Differentiation of the iPSCs into electrically excitable cortical neurons

The iPSCs were generated from 5 controls and 3 *NRXN1α*^+/-^ patients with ASD as described previously [30]. The proliferation potential of the iPSCs was determined using Ki67 and phosphohistone 3, and no significant difference was found between the two groups (Additional file 1: Fig. S1). The iPSCs were differentiated into electrically excitable cortical neurons using dual SMAD inhibition [34, 35]. The iPSCs were treated with LDN193189/SB431542 to induce neural rosettes, at day 20 of differentiation, 82% of cells were stained positive for anti-PAX6 and 87% were positive for anti-NESTIN among the total cells stained by Hoechst [30]. Abundant transcripts of neural progenitor and anterior cortex markers (*PAX6*, *NES*, *FOXG1* and *NEUROG2*) were also detected by qRT-PCR, showing that the majority of day 20 cells were cortical progenitors [30]. The differentiated cultures were evaluated at day 30 by immunocytochemistry using PAX6/NES (neural progenitor markers), Ki67 (proliferative marker) and DCX (newborn neuronal marker), TUJ1 (pan-neuronal marker) and GFAP (astrocyte marker). NRXN1 haploinsufficiency exhibited no significant effect at the early stages of neuronal differentiation (Additional file 1: Fig. S2 ).

**Fig. 1:**
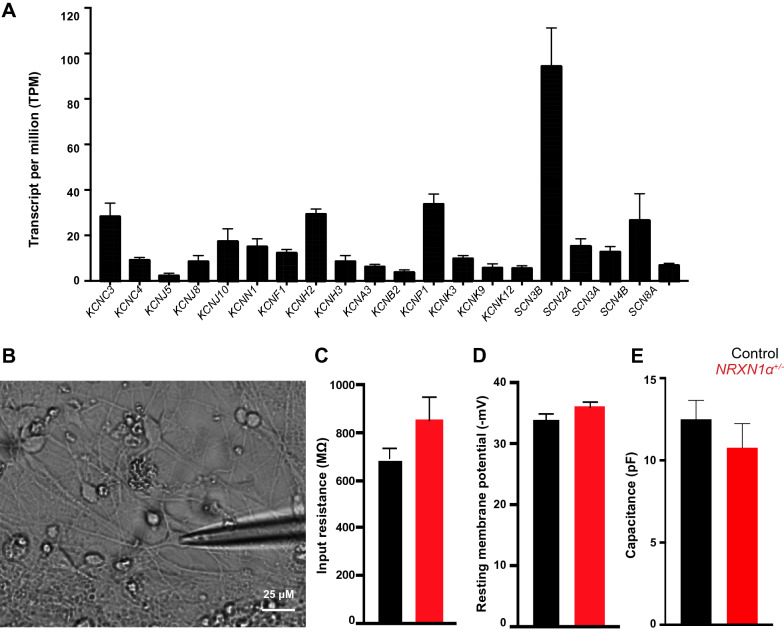
Differentiation of iPSCs into 100-day functional cortical neurons. **A** Quantitative RNASeq data showing expression of various VGKC and VGSC mRNA from the control group as molecular markers for 100 day neurons. **B** Representative image of patch clamp of 100-day neurons. **C** Input resistance, **D** resting membrane potential and capacitance **E** from whole cell recordings of control (n = 45 cells from 29 coverslips) and *NRXN1α*^+/-^ neurons (n = 27 cells on 17 coverslips) showed no significant difference. Data in A, C, D, E are mean ± SEM

The cultures were differentiated to day 100, and most cells were shown to be MAP2^+^ mature (Control 38 ± 1.53; *NRXN1α*^+/-^ 28.67 ± 1.20 mm/mm^2^)neurons with few GFAP^+^ astrocytes (Control 15 ± 4.73; *NRXN1α*^+/-^ 26.67 ± 5.84 mm/mm^2^) detected [35]. Again there was no significant difference in proportions of TUJ1^+^/GFAP^+^ cells between the two groups [35]. Neuronal maturity was confirmed by positive immunostaining of MAP2/SYN1/TUJ1 (Additional file 1: Fig. S3A–C) and by qRT-PCR [30], which did not show gross difference in expression of synaptic density markers. The cultures were shown to contain 23.4% of TBR1^+^ layer VI cortical neurons and 36.2% of CTIP2^+^ layer V-VI cells (Additional file 1: Fig. S3D). The synapse formation was previously evaluated by immunocytochemistry and immunoblotting in the parallel study [30]. High expression levels of voltage-gated sodium channels (VGSCs), voltage-gated potassium channels (VGKCs), voltage-gated calcium channels (VGCCs) and postsynaptic excitatory genes in day-100 cultures were also systematically revealed by RNASeq in the control group (Fig. 1A), which demonstrated the cortical excitatory nature of cells in day 100 cultures.

### ***NRXN1α***^+/-^ alters voltage-gated ion currents

The two groups of day-100 cultures were subsequently compared for their electrophysiology (Additional file 1: Fig. S5). Whole cell recording of iPSC-derived neurons (Fig. 1B) showed no significant difference in the input resistance between control (688.00 ± 46.36 MΩ) and *NRXN1α*^+/-^ neurons (852.00 ± 96.64 MΩ, *p* = 0.21) or resting membrane potential (control − 34.11 ± 0.90 mV; *NRXN1α*^+/-^ − 35.95 ± 0.84 mV, *p* = 0.24), suggesting that there was no significant difference in their passive membrane properties (Fig. 1C, D). Spontaneous APs were detected under current clamp mode with zero current applied (Additional file 1: Fig. S5A–C). The spontaneous EPSCs were also recorded as a measure of synaptic property (Additional file 1: Fig. S5 D–L), and no significant difference was observed between two groups of neurons.

**Fig. 2:**
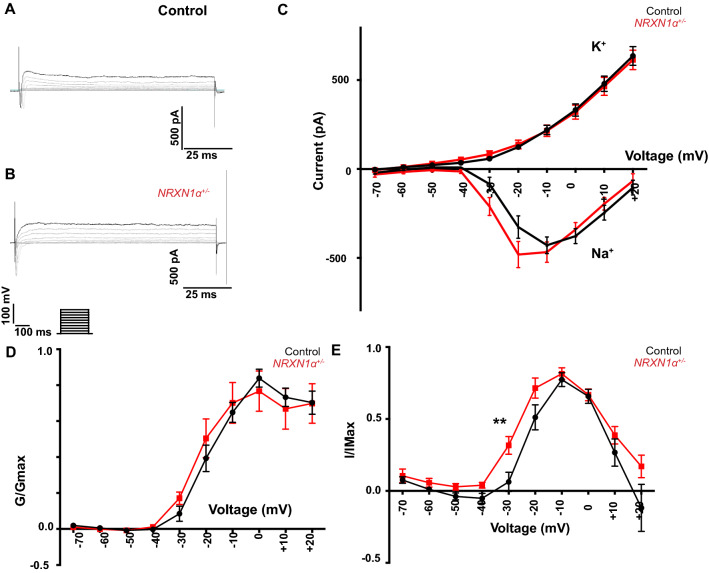
*NRXN1α*^+/-^ deletion significantly impairs voltage-gated Na^+^ currents. Representative traces of voltage clamp recording from **A** control (n = 45 cells from 29 coverslips) and from **B** *NRXN1α*^+/-^ (n = 27 cells on 17 coverslips) iPSC-derived neurons, showing fast inward Na^+^ currents followed by slow outward K^+^ currents, in response to different voltage step applied. **C** Average Na^+^ and K^+^ currents from both controls (black) and *NRXN1α*^+/-^ (red) neurons, showing significant increases of the inward Na^+^ in a voltage-dependent manner in the *NRXN1α*^+/-^ neurons. **D** The data from panel C were normalized to maximal Na^+^ (G/Gmax) **E**, and also normalized to Na^+^ conductance (I/Imax) and plotted against voltage steps, with significant increase at − 30 mV (** *p* < 0.002) and no significant change at − 20 mV (*p* = 0.23). Data were presented as mean ± SEM. Statistical significance (** *p* < 0.01) were evaluated using Man Whitney U test or repeated measure ANOVA

The majority (96%) of patched cells in 100-day cultures produced voltage-dependent current, which were increased with the voltage step applied (− 70 mV to + 20 mV, Fig. 2A, B), and abolished by Na^+^ (TTX) or K^+^ (TEA) channel blockers, respectively (Additional file 1: Fig. S6). There was no significant difference in capacitance between the control (12.50 ± 1.55 pF) and *NRXN1α*^+/-^ (10.77 ± 1.47 pF) neurons (Fig. 1E).

The voltage-gated Na^+^ currents in *NRXN1α*^+/-^ lines (Fig. 2C) were however significantly increased. A repeated measure ANOVA showed that the inward Na^+^ currents of *NRXN1α*^+/- ^neurons were significantly increased in comparison to the voltage change in the control neurons (*p* = 0.026). The normalized Na^+^ conductance (I/Imax, Fig. 2E) were significantly higher in *NRXN1α*^+/-^ cells (*p* = 0.002) at − 30 mV with no change at Na^+^ maximum conductance (G/Gmax, Fig. 2D, E). These data suggest that *NRXN1α*^+/-^ deletions are associated with increased neuronal excitability.

### ***NRXN1α***^+/-^ alters neuronal excitability

**Fig. 3:**
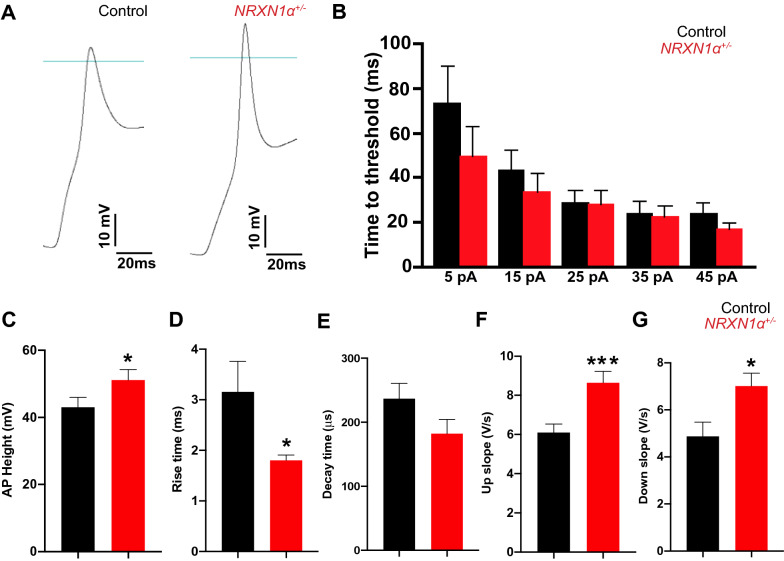
*NRXN1α*^+/-^ deletions significantly alter the electrical excitability of iPSC derived neurons. **A** A representative evoked AP trace from a control and *NRXN1α*^+/-^ neuron. Blue line indicates 0 mV baseline. **B** The time to reach AP firing threshold was decreased as the injected currents increased in both types of cells. **C** The average amplitude of evoked AP was significantly increased in *NRXN1α*^+/-^ neurons. While AP rise time **D** showed significant increase in *NRXN1α*^+/-^ neurons, AP decay time showed no significant impairment between the two groups (*p* = 0.07) **E**. Both the depolarization up slope **F** and repolarization down slope **G** were significantly increased in *NRXN1α*^+/-^ neurons. Data were Mean ± SEM. Statistical significance (*p < 0.05, ***p < 0.001) were evaluated using Mann Whitney U test

VGSCs and VGKCs are known to drive spatial and temporal dynamics of APs in neurons. The effects of *NRXN1α*^+/-^ in voltage-gated Na^+^ and K^+^ currents were subsequently investigated in evoked AP kinetics (Fig. 3A). There was no change in AP threshold voltage between control and *NRXN1α*^+/-^ neurons (Fig. 3B, p = 0.44). The average AP amplitude was 43.01 ± 2.93 mV in controls and increased by 21.47% in *NRXN1α*^+/-^ neurons (51.14 ± 3.15 mV, *p* = 0.03, Fig. 3C). There was also a significant difference in AP rise time with 3.12 ± 0.60 ms in controls and 1.80 ± 0.10 ms in *NRXN1α*^+/-^ neurons (*p* = 0.01, Fig. 3D). However, there was a trend but insignificant decrease in repolarization decay time (*p* = 0.07, Fig. 3E). The AP up slope was 6.10 ± 0.43 V/s in controls, and significantly higher in *NRXN1α*^+/-^ neurons (8.65 ± 0.58 V/s, *p* = 0.0004, Fig. 3F). The average repolarization slope was also significantly increased in *NRXN1α*^+/-^ neurons with 4.88 ± 0.60 V/s in controls and 7.02 ± 0.255 V/s in *NRXN1α*^+/-^ neurons (*p* = 0.02, Fig. 3G). Together these data demonstrated a characteristic of increased excitability of *NRXN1α*^+/-^ neurons in comparison to control neurons.

### Altered VGKC activity and glutamatergic synapses in ***NRXN1α***^+/-^ neurons

**Fig. 4:**
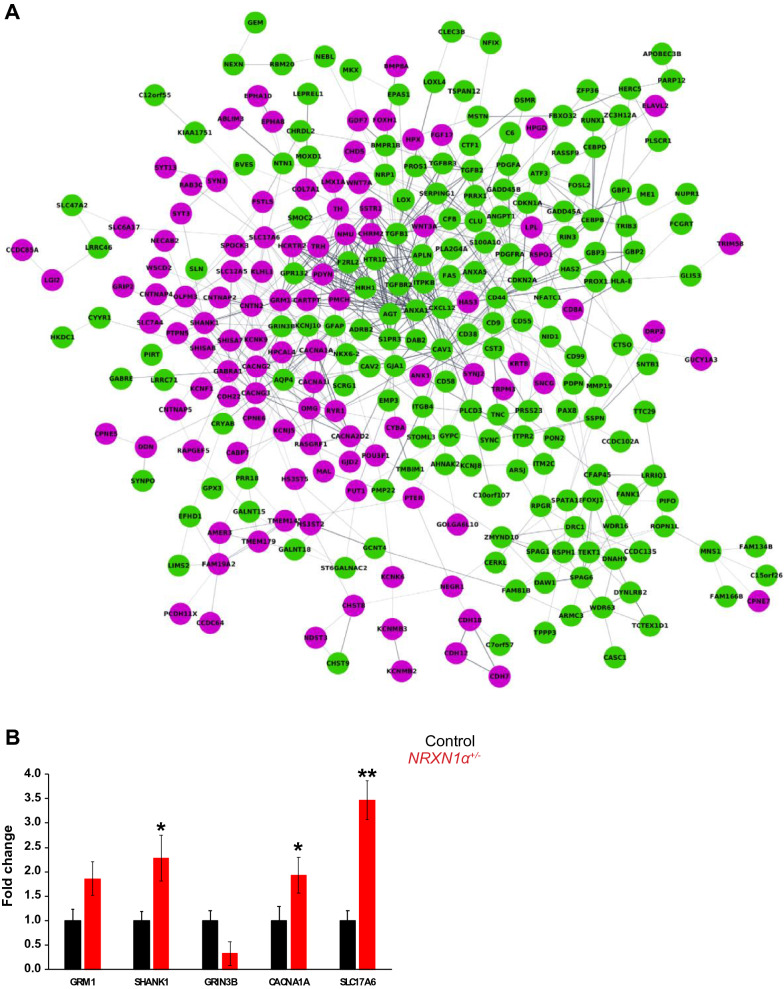
Overview of differential expressed genes in *NRXN1a*^+/-^ ASD neurons and qRT-PCR validation. **A** An overview of the connected protein–protein interaction network of *NRXN1a*^+/-^ targets, showing 269 down-regulated (in green) and 156 upregulated genes (in purple). All DEGs are FDR < 0.05, TPM > 2, either < 50% reduction (in green) or > twofold increase (in purple). **B** Validation of the RNASeq by qRT-PCR in five control (02VC1, 3VC2, 3VCX1, 4C3, 4CX1) and three ASD (ND1C1, ND2C11, ND4-1C1) lines, which showed reduction of *GRIN3B* (0.33x) and the upregulation of *GRM1* (1.86x, *p* = 0.18), *SHANK1* (2.28x, *p = 0.05), *CACNA1A* (1.93x, *p = 0.05) and *SLC17A6* (3.47x, **p = 0.01) respectively

We next performed RNA sequencing on day-100 *NRXN1α*^+/-^ and control neurons. A total of 27,163 transcripts were quantitatively sequenced and 425 DEGs (Additional file 1: Table S1) were identified with FDR < 0.05, TPM > 2, > 50% decrease or > twofold increase in *NRXN1α*^+/-^ neurons (Fig. 4A). We validated expression of limited DEGs by qRT-PCR. Whereas *GRIN3B* expression was reduced to 0.33-fold in the ASD group, the *GRM1, SHANK1, CACNA1A* and *SLC17A6* expression were upregulated by 1.86 (*p* = 0.18), 2.28 (*p* = 0.05), 1.93 (*p* = 0.05) and 3.47-fold (*p* = 0.01), respectively in 100-day neurons derived from ASD iPSCs (Fig. 4B).

**Fig. 5:**
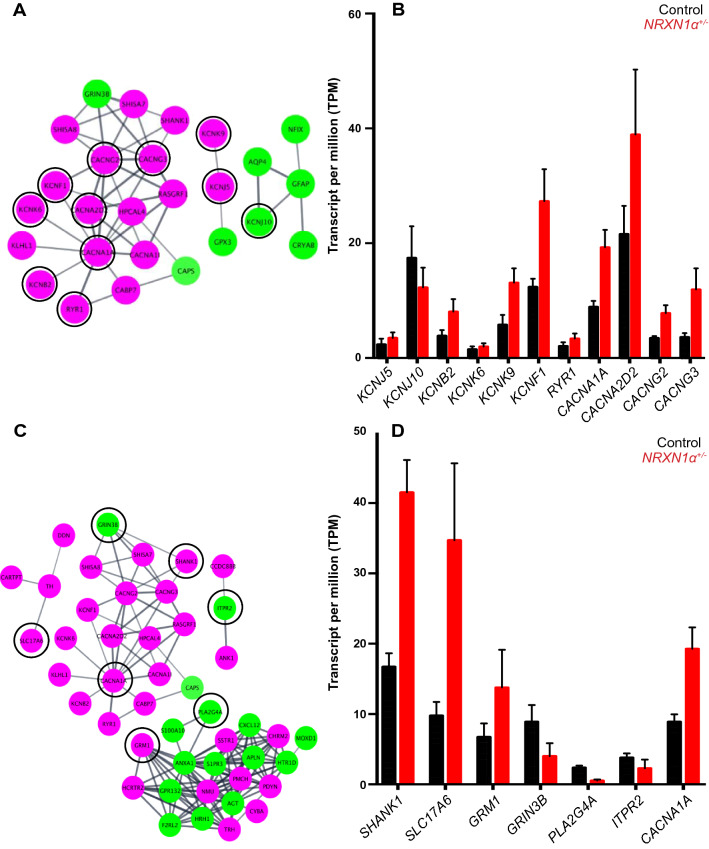
The expression of VGKCs and glutamatergic synapse are significantly dysregulated in *NRXN1a*^+/-^ ASD neurons. **A** Significantly disturbed VGKCs (in back circle) were identified by “*Molecular Function* (*GO*)” group with their relative expression level expressed in TPM **B**. **C** Networks clustered by Cytoscape program with proteins associated with glutamatergic synapse in black circle (FDR = 0.003) from *KEGG* pathway (STRING KEGG pathway). **D** RNASeq data showing relative mRNA expression (in TMP) of the glutamatergic pathway members which were significantly dysregulated in *NRXN1a*^+/-^ neurons

STRING analyses of the 269 upregulated and 156 downregulated DEGs identified significant disrupted pathways of synapses and ion channels, which included Voltage-gated cation channel activity (GO: 0022843, FDR = 5.42E−4) and Ligand-gated ion channel activity (GO:0015276, FDR = 0.025) in the “*Molecular Function (GO)*” analyses, and Ion channel complex (GO:0034702, FDR = 8.17E−6) and Voltage-gated potassium channel complex (GO:0008076, FDR = 0.004) in the “*Cellular Component* (*GO*)”. Among the 14 gated ion channels, *KCNK6* (2.2x)*, KCNK9* (2.3x)*, KCNK12* (2.2x), *KCNF1* (2.1x)*, KCNB2 (*2.1x)*, KCNJ5* (2.9x) and KCNMB2 (2.4x) were significantly upregulated (Fig. 5A, B).

Meanwhile, “Synaptic transmission” was identified as the most significant pathway in “*Biological Processes* (*GO*)*”* with 40 DEGs involved (FDR = 4.05E-09). Similarly, 27 genes were involved in altered “Synapse” in “*Cellular Processes* (*GO*)*”* (FDR = 0.003). The glutamatergic synapse (FDR = 0.003) was a significant “*KEGG*” pathway (Fig. 5C), and the eleven DEGs in the glutamatergic synapse pathway included glutamatergic receptors and vesicular transporters (*GRIN3B* (0.47x)*, GRM1* (2.4x)*, SHANK1* (2.0x)*, SLC17A6* (3.6x)*, CACNA1A* (0.2.0x)*, ITPR2* (0.42x) and *PLA2G4* (0.21x) (Fig. 5D). There was also a significant disruption of Ionotropic glutamate receptor complex including *SHANK1* (2.0x)*, GRIN3B* (0.47x)*, CACNG2* (2.1x) and *CACNG3* (3.3x) in “*Cellular Processes* (*GO*)*”.* In addition, *SCN8A*, *ANK1* and *ANK3* related to AP initiation and propagation at the distal axon initial segment (AIS) were upregulated by 1.5-, 2.4- and 1.5-fold, respectively. The transcriptome analyses thus revealed molecular processes that are accountable for alterations in AP depolarization, accelerated repolarization and hyper-excitability in *NRXN1α*^+/-^ ASD neurons.

## Discussion

*NRXN1*^+/-^ deletions are associated with neurodevelopmental disorders including ASD and epilepsy. Despite its importance and diverse clinical symptoms, how *NRXN1α* deletions affect human neuronal function are largely unknown. As a first step to address this, we derived iPSCs from controls and ASD patients with *NRXN1α*^+/-^, and differentiated them into day-100 cortical neurons. The *NRXN1α*^+/-^ neurons exhibited increased Na^+^ current, higher amplitude of evoked AP, shortened AP rise time, and accelerated depolarization and repolarization slope. Consistent with the electrophysiological changes, there was a significant upregulation of ion channels and glutamatergic synapse genes (*GRM1, SHANK1, SLC17A6, CACNA1A*) in the *NRXN1α*^+/-^ transcriptome, which supports an increased excitability in the ASD neurons. The observed phenotype could be resulted from haplo-insufficiency of *NRXN1α*^+/-^. However, our current RNASeq was unable to reveal isoform alterations and we could not rule out the possibility of gain-of-function as recently suggested [29].

The hyper-excitability phenotype is consistent with the clinical observation of the subjects in the current study, and two of three ASD probands have a history of seizures. In fact, ASD is known to have 30% comorbidity with epilepsy or vice versa, and altered cortical neuronal excitability has been proposed as an important neurological hallmark for ASD [36]. The observed phenotype also fits with the role of *NRXN1α* in both excitation/inhibition, and deletion of *NRXN1α* may lead to net reduction of inhibition. Furthermore, compound *NRXN1α* deletions were reported in patients with more complicated presentations, such as Pitt-Hopkins-like Syndrome displaying severe mental retardation, autistic behavior, epilepsy and breathing anomalies [37], and in patients with ASD, mental retardation and epilepsy [38]. Compound deletions affecting *NRXN1α* on one allele and both *NRXN1α/NRXN1β* on the second allele were identified in two sisters with severe early onset epilepsy, profound developmental delay, gastroesophageal reflux disease, constipation and early onset puberty [39]. Although the cases are limited, these studies suggest that mutations on both *NRXN1α* alleles are likely associated with greater severity, and ASD/epilepsy are common phenotype in these patients.

In line with increased excitability phenotype, our transcriptome analysis also uncovered a significant upregulation of *GRM1*, *SHANK1, SLC17A6, CACNA1A* and downregulation of *GRIN3B, ITPR2,* and *PLA2G4A* in the *NRXN1α*^+/-^ neurons. The majority of these genes are involved in excitatory synapses. For example, *SLC17A6* encodes VGLU2 for presynaptic glutamate uptake, and dysregulation in ionotropic glutamatergic receptor complex including CACGN2, CACGN3, SHANK1 and GRIN3B further supports a disruption in excitatory glutamatergic signalling. In addition, the mean expression levels of sodium voltage-gated channels *SCN2A*, *SCN2B*, *SCN3A* and *SCN3B* were upregulated by 1.64, 1.45, 2.00 and 1.58-fold, respectively, in the patient group. Although they did not reach the stringent shortlisting criteria individually, they might contribute to the increased action potential collectively.

Interestingly, the transcriptome data also showed 1.5-fold increase (FDR = 1.23E−03) of *SCN8A* (encoding the channel Nav1.6) in *NRXN1α*^+/-^ neurons. This fits with gain-of-function of *SCN8A* previously reported in patients with ASD and early infantile epileptic encephalopathies [40], and six SCN8A variants with disrupted channel inactivation were also identified from 277 patients with ASD, epilepsy, intellectual disability and neuromuscular disorders [41]. Nav1.6 is the main VGSC clustered at the distal AP initiation site (AIS) and plays a major role in the initiation and propagation of cortical pyramidal neurons [42, 43]. In mice, *Scn8a* was upregulated in the hippocampus following epilepticus, whereas *Scn8a* knockdown prevented the induction of spontaneous seizures [44]. An increase in the available pool of sodium channels may therefore partially explain a more rapid rise of AP in *NRXN1α*^+/- ^neurons.

At the AIS subdomain and nodes of Ranvier, Nav1.6 interacts with ankyrins [45]. Ankyrin mutations were previously reported in neurodevelopmental and psychiatric disorders, and ankyrin-G knockout mice displayed severe deficits in AP [46, 47]. In this study, *ANK3* (1.5-fold, FDR = 4.24E−02) and *ANK1* (2.4-fold, FDR = 1.37E−05) were upregulated in *NRXN1α*^+/-^ neurons, and increased abundance of SCN8A-ANK3 complex might contribute to the alteration in AP depolarization dynamics and increased excitability in the *NRXN1α*^+/-^ neurons.

Additionally, a number of VGKCs including *KCNB2*, *KCNF1*, *KCNK6*, *KCNK9* and *KCNJ5* are significantly upregulated in the *NRXN1α*^+/-^ transcriptome, and gain of function of VGKCs has been identified in neurodevelopmental disorders including epilepsy [48–50]. VGKC channels are known as major contributing factors to neuronal membrane potential and stabilization of excitability. This correlates with the accelerated repolarization slope in *NRXN1α*^+/-^ neurons, as accelerated slope may reflect the increased driving force affecting the movement of K^+^ (to stabilize membrane potential), which arises from the higher level of membrane depolarization due to the increased Na^+^ current. However, it is not clear at this stage how *NRXN1α* deletions lead to elevated VGKC expression. In relation to this, a neurexin family member, CASPR2, is essential for AP propagation by controlling the localization of Kv1.1, Kv1.2 and TAG-1 complex [51]. It remains to be explored if NRXN1α can directly modulate these VGKCs by physical interactions.

It is worth to note that the current hyper-excitability phenotype of *NRXN1α*^+/-^ ASD neurons appears to differ from the phenotype reported for *Nrxn1*^+/-^ or *Nrxn1*^*−/−*^ mouse neurons (which showed no significant phenotype) or for human H1 ESC-derived *NRXN1*^+/-^ cortical neurons (which displayed reduced mEPSCs) [26]. This could arise from genetic differences as the mutant ESCs were *NRXN1α*^+/-^/*NRXN1β*^+/-^ or *NRXN1α*^+/-^/*NRXN1β*^+/-^/*NRXN1γ*^+/-^ [26], whereas the ASD subjects in the current study were *NRXN1α*^+/-^. There could also be unknown secondary factors in the patient genome which might not be present in H1 ESC genome. The difference to mouse phenotype could partially be due to species difference in sensitivity to *NRXN1* deficiency as previously suggested [26]. Furthermore, NRXN signalling was shown to be complex, and the same *Nrxn1α* lesion in mouse produced different phenotypes in different neurons or different synapses [52, 53].

The current study demonstrates that ASD patient-derived neurons with *NRXN1α*^+/-^ showed impairment in non-synaptic neuronal properties. The hyper-excitability could result in an increase in Ca^2+^ influx at the synaptic terminals, and an increased spontaneous calcium transients was indeed observed in the previous study [30]. This is supported by similar phenotype in iPSC-derived excitatory neurons from ASD probands carrying *CNTN5*^+/-^ and *EHMT2*^+/-^ [54]. While research on patient-derived samples enables a good understanding of *NRXN1* function in human neurons for pleiotropic clinical presentations, there are limitations of the study. Although NRXN1α haploinsufficiency is shown to have no significant effect on iPSC proliferation, early neuronal differentiation, or 100-day neuronal maturity, this does not completely eliminate potential effect on developmental pace. In 100-day cultures, not all neurons have the same maturity or are same neuronal subtype. Neurons may therefore be subtyped before/after patch clamping, and RNAseq may be carried out at single cell level to evaluate contribution of neuronal subtype to the observed phenotype in the future studies. It is also important to create multiple isogenic lines carrying *NRXN1* isoform lesions on the same genetic background and to validate their influence on neuronal excitation/inhibition in the follow-up studies, although it is challenging to precisely rescue the genetic defects in patients’ iPSC lines due to hundreds of kilobase pair DNA are deleted.

## Conclusions

*NRXN1* deletion is a major rare risk factor in ASD. In this study NRXN1*α*^+/-^ neurons derived from iPSCs of three ASD patients were shown to display an increase in excitability, which was well co-related with our recent evidence of increased calcium transients [51]. This is the first demonstration that human ASD *NRXN1*α^+/-^ deletions can lead to neuronal hyper-excitability. The future therapeutic development in neurodevelopmental disorders will require a personalized medicine approach targeting different underlying mechanisms of many underlying variants. The *NRXN1α*^+/-^ iPSCs such provide a human cell model for our further understanding of molecular/cellular mechanisms and NRXN1 signalling pathways which are associated with ASD.

## Methods

### Participants

Five *NRXN1α*^+/-^ iPSC lines from three ASD patients (ND1, ND2, ND4-1) were investigated in this study [28]. All patients were diagnosed with ASD by the Autism Diagnostic Interview-Revised and the Autism Diagnostic Observational Schedule. ND1 carries de novo* NRXN1α*^+/-^ deletion on exons 6–15 (chr2:50711687–51044633, 332,946 bp, hg19). At the time of biopsy, ND1 was an 8-year-old non-verbal ASD male with a history of infantile seizures, severe intellectual disability, developmental delay, self-injurious and aggressive behavior. ND2 harbors *NRXN1α*^+/-^ deletion in exon 1–5 (Chr2:51120335–51360666, 240,331 bp, hg19). He was a 20-year ASD male with language delay and an IQ of 78 at age 11 but attended mainstream education. He has a reported family history of ASD affecting a grandfather and a cousin, and a history of language delay in one parent. ND4-1 carries a paternally inherited *NRXN1α*^+/-^ deletion on exon 1–5 (chr2:50983186–51471321, 488,135 bp, hg19). She was an 18-year female with a diagnosis of Asperger’s Syndrome, a history of seizures, social anxiety, psychosis and mild intellectual disability with an IQ of 69. Her father and paternal aunt had adult-onset seizures, and her paternal grandmother was institutionalized. She has a brother who carries the same paternally inherited deletion with oppositional behavior and sub-clinical ASD symptoms.

Six control iPSC lines from five healthy donors were used in the study. 1C was from a 4-year-old healthy sibling of ND1, 4C was from a 19-year-old male sibling of ND2, 2V was from a 20-year-old female undergraduate, the 3V was from a 21-year-old male undergraduate, and the NCRM-1 was an iPSC line from NIH which was derived from a newborn boy. The study was carried out with full ethical approval granted by Galway University Hospital and St. James’s/Tallaght University Hospital Clinical Research Ethics Committees.

### iPSC derivation

Volunteers were recruited with consent for the study and skin biopsies (3 mm) were obtained by named clinicians with local anesthesia. Individual punch was minced, adhered to scratched surface of 6-well plates and cultured at 37 °C with 5% CO_2_ in high glucose DMEM supplemented with 10% FCS, 1% penicillin/streptomycin and 1% NEAA. The medium was renewed every 2–3 days. Low passage fibroblasts were reprogrammed under manufacturer’s instructions (Merck-Millipore, SCR510; ThermoFisher Scientific, A15960). The iPSCs were characterized for the pluripotency markers of alkaline phosphatase, NANOG, OCT4, SOX2, SSEA4, TRA-1–60, TRA-1–81, and markers for tri-germ layer differentiation, TUJ1/ASM/AFP, and SNP karyotyping.

### Confirmation of CNVs

Genomic DNA was isolated from the fibroblasts and derived iPSC lines using Qiagen DNeasy Blood & Tissue kit (69,504, Qiagen). Genotype was performed on an Illumina 1M SNP array at UCD. Quality control analysis showed that all samples had call rates > 99%. CNV analysis was undertaken using PennCNV. Short CNVs (i.e. containing < 10 SNPs and/or < 50 kb in length) were excluded to prevent false positive CNVs. The *NRXN1α*^+/-^ deletions in patients were confirmed by the SNP array.

### Neuronal differentiation

The iPSCs were seeded in 6-well plates at 45,000–50,000 cells/cm^2^, grown to ~ 80% confluency in E8 medium (ThermoFisher Scientific, A1517001), and differentiated into neural rosettes for 10–12 days in N2B27 medium (ThermoFisher Scientific) with 100 nM LDN193189 (Stem Cell technologies, #72,102) and 10 nM SB431542 (Sigma, S4317). Half of the medium was renewed daily with fresh SB431542/LDN193189 added. Neural rosettes were passaged with half of the medium changed every 2nd day for 10 days and then passaged onto Poly-D-Lysine/laminin-coated 12-well plates, or 15-mm round coverslips (ThermoFisher Scientific, 12362138), or ibidi 8-well chambers for terminal differentiation. Cells were fed with N2B27 (w/o vitamin A), every 2–3 days for 6 days and then in N2B27 Plus vitamin A until day 100. They were finally processed for patch clamping, immunocytochemistry, immunoblotting or RNA sequencing, respectively.

### Electrophysiology

Whole cell patch clamp configuration was used to record from day-100 neurons (control n = 54, patient n = 39, and median of 10 cells recorded per line for both control and patients). All recordings were performed in warm extracellular bath solution, containing 140 mM NaCl (Sigma-Aldrich 71,387), 5 mM KCl (Sigma-Aldrich P9333), 2 mM CaCl_2_ (Sigma-Aldrich C5670), 2 mM MgCl_2_ (Sigma-Aldrich M8266), 10 mM HEPES sodium salt (Sigma-Aldrich H7006) and 10 mM glucose at pH 7.4. Single cells were selected for recordings based on a pyramidal morphology, bright/clear cell body and each with > 3 neurites. Images were taken under Zeiss Axiovert 200 (40X).

Patch pipettes were pulled on borosilicate glass capillaries (Harvard apparatus GC150TF-7.5) using a Zeitz DMZ puller (Werner Zeitz, Germany), and filled with intracellular solution containing 123 mM K-gluconate, 10 mM KCL, 1 mM MgCl2, 10 mM HEPES potassium salt (Sigma-Aldrich H0527), 1 mM EGTA, 0.1 mM CaCl2, 1.5 mM ATP magnesium salt (Sigma-Aldrich A9187), 0.2 mM GTP sodium salt hydrate (Sigma-Aldrich 51,120) and 4 mM glucose with resistance of 4.0–5.5 MΩ. Recordings were made using the EPC10 patch clamp amplifier from HEKA. Voltage-dependent Na^+^ and K^+^ currents were recorded in voltage clamp mode from a holding potential of − 70 mV; voltage step depolarization was applied up to + 20 mV in 10 mV increments and recorded for 200 mSec. Additional recordings were performed in the presence of 1 µM TTX (Alomone labs, T-550) or 10 mM TEA (Sigma Aldrich, 140,023) to block Na^+^ and K^+^ currents, respectively. All data were recorded unfiltered for voltage and current clamp recording at 50.0 kHz and 20.0 kHz, respectively. Series resistance was 90% compensated for using the built-in circuitry of the amplifier and monitored throughout, only recordings that had a stable series resistance of < 20MΩ were included for analysis (control n = 45, patient n = 27, and median of 6 cells recorded per line for both control and patients).

Intrinsic firing properties of neurons were recorded in current clamp mode, with step current injections in 10 pA increments from a holding value of − 5 pA up to + 45 pA, for 500 mSec. Spontaneous AP was recorded in current clamp mode at zero current injection (resting membrane potential) just after whole cell configuration achieved. Data were analysed using patchmaster and fitmaster (HEKA). The AP threshold was measured in Igorpro as a change in slope on each AP. AP amplitude was measured as the peak voltage from the point of threshold in patchmaster. Rise time of AP during depolarization was measured as the time between 20 and 80% of the rising edges of the AP. Depolarization up slope defines as slope between the two levels on the rising edges of the AP depolarization. Repolarization down slope was measured as a slope between the two levels of the falling edges of the AP repolarization. Currents were normalized to maximum conductance (G_max_ = I/(V-V_r_) and plotted against each voltage step. The conductance (G) was calculated by dividing each voltage with the reversal potential (V_r_).

### Immunocytochemistry

Cells were washed with PBS and fixed in 4% paraformaldehyde (Santa Cruz 30,525–89-4) for 20 min at room temperature (RT). They were then washed 3 times in PSB for 5 min before blocking for 1 h at RT in PBS with 0.2% BSA (Sigma Aldrich A2153) and 0.1% triton-X100. Cells were then incubated at 4º C overnight with primary antibody anti-MAP2 (Abcam Ab32454, 1:200), SYN1 (Abcam Ab8, 1:1000), TUJ1 (Abcam Ab78078, 1:1000), CTIP2 (Abcam Ab18465, 1:400) or TBR1 (Abcam Ab183032, 1:400) in blocking solution. Next day cells were washed 3 times in PBS and incubated for 1 h at RT with appropriate fluorophore conjugated secondary antibodies (Cell Signaling, 4409S, or 4412S, 1:1000). Cells were washed 3 times in PBS and then imaged using Andor confocal microscope. Image J was used to quantify the images which were normalized by DAPI-positive nuclei.

### Quantitative RT-PCR

Media were aspirated, cells washed and RNA extracted using RNeasy Mini kit (Qiagen 74,104) following the manufacturer’s instructions. RNA concentration and purity were measured using Nanodrop. 1 µg RNA reversely transcribed into cDNA (Qiagen QuantiTect Reverse Transcription kit 205,311), and qRT-PCR was carried out in triplicates with 10 ng cDNA template per reaction. The resulted cycle threshold (Ct) value were normalized to that of *GAPDH*. The relative mRNA expressions were calculated as 2 ^–dCt^ to *GAPDH* or 2 ^–ddCt^ using the average dCt of a fibroblast or an iPSC line.

### Transcriptomic analysis

Quantitative RNA sequencing was performed by BGI as described previously [55, 56] on iPSC-derived cortical neurons from 6 control lines (2VC1, 3VCX1, 3VC2, 4C3, 4CX1, NCRM-1) of 4 healthy donors and 4 *NRXN1α*^+/-^ lines (ND1C1, ND2C11, ND2CX1, ND4-1C2) from 3 ASD patients. A total of 27,163 transcripts were mapped to GRCH37/hg19. Transcript abundance was quantified from the FASTQ files in Kallisto (v0.43.1) and presented as Transcripts Per Million (TPM). The control and patient groups were analyzed with false discovery rate (FDR) and adjusted multiple P value using the DESeq2 package in R. Using FDR < 0.05, 1175 DEGs was identified, and filtered with TPM > 2, > twofold increase or > 50% decrease, which resulted in 269 significantly down-regulated and 156 upregulated genes. The 425 DEGs were analyzed by STRING and Gene Set Enrichment Analysis (GSEA) for *NRXN1*α^+/-^ pathways. The KEGG pathway analysis were obtained via KEGG enrichment results from STRING [57, 58]. The qRT-PCR was carried out to validate the expression of *GRM1, SHANK1, GRIN3B, CACNA1A* and *SLC17A6* genes with mRNA extracted from 100-day neurons of five control (02VC1, 3VC2, 3VCX1, 4C3, 4CX1) and three ASD (ND1C1, ND2C11, ND4-1C1) iPSC lines. The PCR primers included *GRM1For* (*5′-AGTGAGCTGCTGCTGGATTTG-3′*), *GRM1Rev* (*5′-TGCTCCACTCAAGATAGCGCA-3′,*149 bp)*, SHANK1For* (*5′-TTTGCCACTGAGTCGAGCTTC-3′*), *SHANK1Rev* (*5′-ACATCTTCTGCCGCACCGATA-3′,* 125 bp)*, GRIN3BFor* (*5′-CAACCTGTCCGAGTTCATCAG-3′*), *GRIN3BRev* (*5′-CGAAGTGGTAGATGCTCATCTG-3′,* 140 bp), *CACNA1AFor* (*5′-AAGGATCGGAAGCATCGACAG-3′*), *CACNA1ARev (5′-CTTCCACTTACGGAACTACTGC-3′,* 197 bp), *SLC17A6For (5′-TTTGGCATGGAAGCCACACTG-3′*), *SLC17A6Rev* (*5′-TCCTGACAATGTGCCAACACC-3′,* 195 bp), *GAPDHFor (5′-CACCAGGTGGTCTCCTCTGA-3′*) and *GAPDHRev (5′-GGTGGTCCAGGGGTCTTACT-3′*, 189 bp).

### Statistics

All data were expressed as mean ± SEM. All data were tested for normality using Shapiro–Wilk normality test. Statistical analysis was performed using student t-test, Mann Whitney U and repeated measure ANOVA test with a *p* < 0.05.

## Supplementary Information

**Additional file 1.** Additional figures and tables.

## Data Availability

The datasets used and/or analysed during the current study are available in the European genome-phenom archive (EGA) repository with accession number of EGAS00001005536.

## Abbreviations

AP: Action potential
ASD: Autism spectrum disorder
DEGs: Differentially expressed genes
E/I: Excitation/inhibition
iPSC: Induced pluripotent stem cell
NRXN: Neurexin
VGCC: Voltage gated calcium channel
VGKC: Voltage gated potassium channel
VGSC: Voltage gated sodium channel

## References

[1] Amaral DG, Schumann CM, Nordahl CW (2008). Neuroanatomy of autism. Trends Neurosci.

[2] Hirvikoski T, Mittendorfer-Rutz E, Boman M, Larsson H, Lichtenstein P, Bölte S (2016). Premature mortality in autism spectrum disorder. Br J Psychiatry.

[3] Marshall CR, Noor A, Vincent JB, Lionel AC, Feuk L, Skaug J (2008). Structural variation of chromosomes in autism spectrum disorder. Am J Hum Genet.

[4] Bucan M, Abrahams BS, Wang K, Glessner JT, Herman EI, Sonnenblick LI (2009). Genome-wide analyses of exonic copy number variants in a family-based study point to novel autism susceptibility genes. PLoS Genet.

[5] Wang Z, Gerstein M, Snyder M (2009). RNA-Seq: a revolutionary tool for transcriptomics. Nat Rev Genet.

[6] Ching MSL, Shen Y, Tan W-H, Jeste SS, Morrow EM, Chen X (2010). Deletions of NRXN1 (neurexin-1) predispose to a wide spectrum of developmental disorders. Am J Med Genet B, Neuropsychiatr Genet.

[7] Béna F, Bruno DL, Eriksson M, van Ravenswaaij-Arts C, Stark Z, Dijkhuizen T (2013). Molecular and clinical characterization of 25 individuals with exonic deletions of NRXN1 and comprehensive review of the literature. Am J Med Genet B, Neuropsychiatr Genet.

[8] Viñas-Jornet M, Esteba-Castillo S, Gabau E, Ribas-Vidal N, Baena N, San J (2014). A common cognitive, psychiatric, and dysmorphic phenotype in carriers of NRXN1 deletion. Mol Genet Genomic Med.

[9] Gregor A, Albrecht B, Bader I, Bijlsma EK, Ekici AB, Engels H (2011). Expanding the clinical spectrum associated with defects in CNTNAP2 and NRXN1. BMC Med Genet.

[10] Schaaf CP, Boone PM, Sampath S, Williams C, Bader PI, Mueller JM (2012). Phenotypic spectrum and genotype-phenotype correlations of NRXN1 exon deletions. Eur J Hum Genet.

[11] Dabell MP, Rosenfeld JA, Bader P, Escobar LF, El-Khechen D, Vallee SE (2013). Investigation of NRXN1 deletions: clinical and molecular characterization. Am J Med Genet A.

[12] Møller RS, Weber YG, Klitten LL, Trucks H, Muhle H, Kunz WS (2013). Exon-disrupting deletions of NRXN1 in idiopathic generalized epilepsy. Epilepsia.

[13] Zahir FR, Baross A, Delaney AD, Eydoux P, Fernandes ND, Pugh T (2008). A patient with vertebral, cognitive and behavioural abnormalities and a de novo deletion of NRXN1alpha. J Med Genet.

[14] Wiśniowiecka-Kowalnik B, Nesteruk M, Peters SU, Xia Z, Cooper ML, Savage S (2010). Intragenic rearrangements in NRXN1 in three families with autism spectrum disorder, developmental delay, and speech delay. Am J Med Genet B, Neuropsychiatr Genet.

[15] Vrijenhoek T, Buizer-Voskamp JE, van der Stelt I, Strengman E, Genetic Risk and Outcome in Psychosis (GROUP) Consortium, Sabatti C, et al. Recurrent CNVs disrupt three candidate genes in schizophrenia patients. Am J Hum Genet. 2008;83:504–10. doi: 10.1016/j.ajhg.2008.09.011. 10.1016/j.ajhg.2008.09.011 PMC2561936 18940311

[16] Need AC, Ge D, Weale ME, Maia J, Feng S, Heinzen EL (2009). A genome-wide investigation of SNPs and CNVs in schizophrenia. PLoS Genet.

[17] Rujescu D, Ingason A, Cichon S, Pietiläinen OPH, Barnes MR, Toulopoulou T (2009). Disruption of the neurexin 1 gene is associated with schizophrenia. Hum Mol Genet.

[18] Kirov G, Rees E, Walters JTR, Escott-Price V, Georgieva L, Richards AL (2014). The penetrance of copy number variations for schizophrenia and developmental delay. Biol Psychiatry.

[19] Yan Q, Weyn-Vanhentenryck SM, Wu J, Sloan SA, Zhang Y, Chen K (2015). Systematic discovery of regulated and conserved alternative exons in the mammalian brain reveals NMD modulating chromatin regulators. Proc Natl Acad Sci USA.

[20] Sterky FH, Trotter JH, Lee S-J, Recktenwald CV, Du X, Zhou B (2017). Carbonic anhydrase-related protein CA10 is an evolutionarily conserved pan-neurexin ligand. Proc Natl Acad Sci USA.

[21] Ullrich B, Ushkaryov YA, Südhof TC (1995). Cartography of neurexins: more than 1000 isoforms generated by alternative splicing and expressed in distinct subsets of neurons. Neuron.

[22] Ushkaryov YA, Petrenko AG, Geppert M, Südhof TC (1992). Neurexins: synaptic cell surface proteins related to the alpha-latrotoxin receptor and laminin. Science.

[23] Trotter JH, Hao J, Maxeiner S, Tsetsenis T, Liu Z, Zhuang X (2019). Synaptic neurexin-1 assembles into dynamically regulated active zone nanoclusters. J Cell Biol.

[24] Geppert M, Khvotchev M, Krasnoperov V, Goda Y, Missler M, Hammer RE (1998). Neurexin I alpha is a major alpha-latrotoxin receptor that cooperates in alpha-latrotoxin action. J Biol Chem.

[25] Etherton MR, Blaiss CA, Powell CM, Südhof TC (2009). Mouse neurexin-1alpha deletion causes correlated electrophysiological and behavioral changes consistent with cognitive impairments. Proc Natl Acad Sci USA.

[26] Pak C, Danko T, Zhang Y, Aoto J, Anderson G, Maxeiner S (2015). Human Neuropsychiatric disease modeling using conditional deletion reveals synaptic transmission defects caused by heterozygous mutations in NRXN1. Cell Stem Cell.

[27] Todarello G, Feng N, Kolachana BS, Li C, Vakkalanka R, Bertolino A (2014). Incomplete penetrance of NRXN1 deletions in families with schizophrenia. Schizophr Res.

[28] Al Shehhi M, Forman EB, Fitzgerald JE, McInerney V, Krawczyk J, Shen S (2019). NRXN1 deletion syndrome; phenotypic and penetrance data from 34 families. Eur J Med Genet.

[29] Flaherty E, Zhu S, Barretto N, Cheng E, Deans PJM, Fernando MB (2019). Neuronal impact of patient-specific aberrant NRXN1α splicing. Nat Genet.

[30] Avazzadeh S, McDonagh K, Reilly J, Wang Y, Boomkamp SD, McInerney V (2019). Increased Ca2+ signaling in NRXN1α+/- neurons derived from ASD induced pluripotent stem cells. Mol Autism.

[31] Rubenstein JLR, Merzenich MM (2003). Model of autism: increased ratio of excitation/inhibition in key neural systems. Genes Brain Behav.

[32] Pinto D, Pagnamenta AT, Klei L, Anney R, Merico D, Regan R (2010). Functional impact of global rare copy number variation in autism spectrum disorders. Nature.

[33] Pinto D, Delaby E, Merico D, Barbosa M, Merikangas A, Klei L (2014). Convergence of genes and cellular pathways dysregulated in autism spectrum disorders. Am J Hum Genet.

[34] Chambers SM, Fasano CA, Papapetrou EP, Tomishima M, Sadelain M, Studer L (2009). Highly efficient neural conversion of human ES and iPS cells by dual inhibition of SMAD signaling. Nat Biotechnol.

[35] Shi Y, Kirwan P, Livesey FJ (2012). Directed differentiation of human pluripotent stem cells to cerebral cortex neurons and neural networks. Nat Protoc.

[36] Rubenstein JLR (2010). Three hypotheses for developmental defects that may underlie some forms of autism spectrum disorder. Curr Opin Neurol.

[37] Zweier C, de Jong EK, Zweier M, Orrico A, Ousager LB, Collins AL (2009). CNTNAP2 and NRXN1 are mutated in autosomal-recessive Pitt-Hopkins-like mental retardation and determine the level of a common synaptic protein in Drosophila. Am J Hum Genet.

[38] Duong L, Klitten LL, Møller RS, Ingason A, Jakobsen KD, Skjødt C (2012). Mutations in NRXN1 in a family multiply affected with brain disorders: NRXN1 mutations and brain disorders. Am J Med Genet B, Neuropsychiatr Genet.

[39] Harrison V, Connell L, Hayesmoore J, McParland J, Pike MG, Blair E (2011). Compound heterozygous deletion of NRXN1 causing severe developmental delay with early onset epilepsy in two sisters. Am J Med Genet A.

[40] Ohba C, Kato M, Takahashi S, Lerman-Sagie T, Lev D, Terashima H (2014). Early onset epileptic encephalopathy caused by de novo SCN8A mutations. Epilepsia.

[41] Butler KM, da Silva C, Shafir Y, Weisfeld-Adams JD, Alexander JJ, Hegde M (2017). De novo and inherited SCN8A epilepsy mutations detected by gene panel analysis. Epilepsy Res.

[42] Hu W, Tian C, Li T, Yang M, Hou H, Shu Y (2009). Distinct contributions of Na(v)1.6 and Na(v)1.2 in action potential initiation and backpropagation. Nat Neurosci..

[43] Kole MHP, Stuart GJ (2012). Signal processing in the axon initial segment. Neuron.

[44] Wong JC, Makinson CD, Lamar T, Cheng Q, Wingard JC, Terwilliger EF (2018). Selective targeting of Scn8a prevents seizure development in a mouse model of mesial temporal lobe epilepsy. Sci Rep.

[45] Iqbal Z, Vandeweyer G, van der Voet M, Waryah AM, Zahoor MY, Besseling JA (2013). Homozygous and heterozygous disruptions of ANK3: at the crossroads of neurodevelopmental and psychiatric disorders. Hum Mol Genet.

[46] Zhou D, Lambert S, Malen PL, Carpenter S, Boland LM, Bennett V (1998). AnkyrinG is required for clustering of voltage-gated Na channels at axon initial segments and for normal action potential firing. J Cell Biol.

[47] Jenkins SM, Bennett V (2001). Ankyrin-G coordinates assembly of the spectrin-based membrane skeleton, voltage-gated sodium channels, and L1 CAMs at Purkinje neuron initial segments. J Cell Biol.

[48] Yang Y, Vasylyev DV, Dib-Hajj F, Veeramah KR, Hammer MF, Dib-Hajj SD (2013). Multistate structural modeling and voltage-clamp analysis of epilepsy/autism mutation Kv10.2-R327H demonstrate the role of this residue in stabilizing the channel closed state. J Neurosci..

[49] Lee H, Lin MA, Kornblum HI, Papazian DM, Nelson SF (2014). Exome sequencing identifies de novo gain of function missense mutation in KCND2 in identical twins with autism and seizures that slows potassium channel inactivation. Hum Mol Genet.

[50] Torkamani A, Bersell K, Jorge BS, Bjork RL, Friedman JR, Bloss CS (2014). De novo KCNB1 mutations in epileptic encephalopathy. Ann Neurol.

[51] Poliak S, Salomon D, Elhanany H, Sabanay H, Kiernan B, Pevny L (2003). Juxtaparanodal clustering of Shaker-like K+ channels in myelinated axons depends on Caspr2 and TAG-1. J Cell Biol.

[52] Aoto J, Földy C, Ilcus SMC, Tabuchi K, Südhof TC (2015). Distinct circuit-dependent functions of presynaptic neurexin-3 at GABAergic and glutamatergic synapses. Nat Neurosci.

[53] Chen LY, Jiang M, Zhang B, Gokce O, Südhof TC (2017). Conditional deletion of all neurexins defines diversity of essential synaptic organizer functions for neurexins. Neuron.

[54] Deneault E, Faheem M, White SH, Rodrigues DC, Sun S, Wei W (2019). CNTN5-/+or EHMT2-/+human iPSC-derived neurons from individuals with autism develop hyperactive neuronal networks. Elife..

[55] Liu M, Guan Z, Shen Q, Flinter F, Domínguez L, Ahn JW (2016). Ulk4 regulates neural stem cell pool. Stem Cells.

[56] Liu M, Guan Z, Shen Q, Lalor P, Fitzgerald U, O’Brien T (2016). Ulk4 is essential for ciliogenesis and CSF flow. J Neurosci.

[57] Szklarczyk D, Gable AL, Lyon D, Junge A, Wyder S, Huerta-Cepas J (2019). STRING v11: protein-protein association networks with increased coverage, supporting functional discovery in genome-wide experimental datasets. Nucleic Acids Res.

[58] Shannon P, Markiel A, Ozier O, Baliga NS, Wang JT, Ramage D (2003). Cytoscape: a software environment for integrated models of biomolecular interaction networks. Genome Res.

