# Supplementary material for: NRXN1α+/- is associated with increased excitability in ASD iPSC-derived neurons
Source: BMC Neurosci. 2021 Sep 15;22:56. doi: 10.1186/s12868-021-00661-0 (PMC8442436; doi:10.1186/s12868-021-00661-0)
Supplement: Supplementary file 1 — Additional file 1. Additional figures and tables. [file 12868_2021_661_MOESM1_ESM.docx]

***NRXN1***α***^+/-^* is associated with increased excitability in ASD iPSC-derived neurons**

Sahar Avazzadeh^1^, Leo R. Quinlan^2^, Jamie Reilly^1^, Katya McDonagh^1^, Amirhossein Jalali^3^, Yanqin Wang^1,4^, Veronica McInerney^5^, Janusz Krawczyk^6^, Yicheng Ding^1^, Jacqueline Fitzgerald^7^, Matthew O’Sullivan^7^, Eva B. Forman^8^, Sally A. Lynch^9^, Sean Ennis^10^, Niamh Feerick^11^, Richard Reilly^11^，Weidong Li^12^, Xu Shen^13^, Guangming Yang^14^, Yin Lu^15^, Hilde Peeters^16^, Peter Dockery^17^, Timothy O’Brien^1^, Sanbing Shen^1,18^*, Louise Gallagher^7^

Additional Figures and tables

**
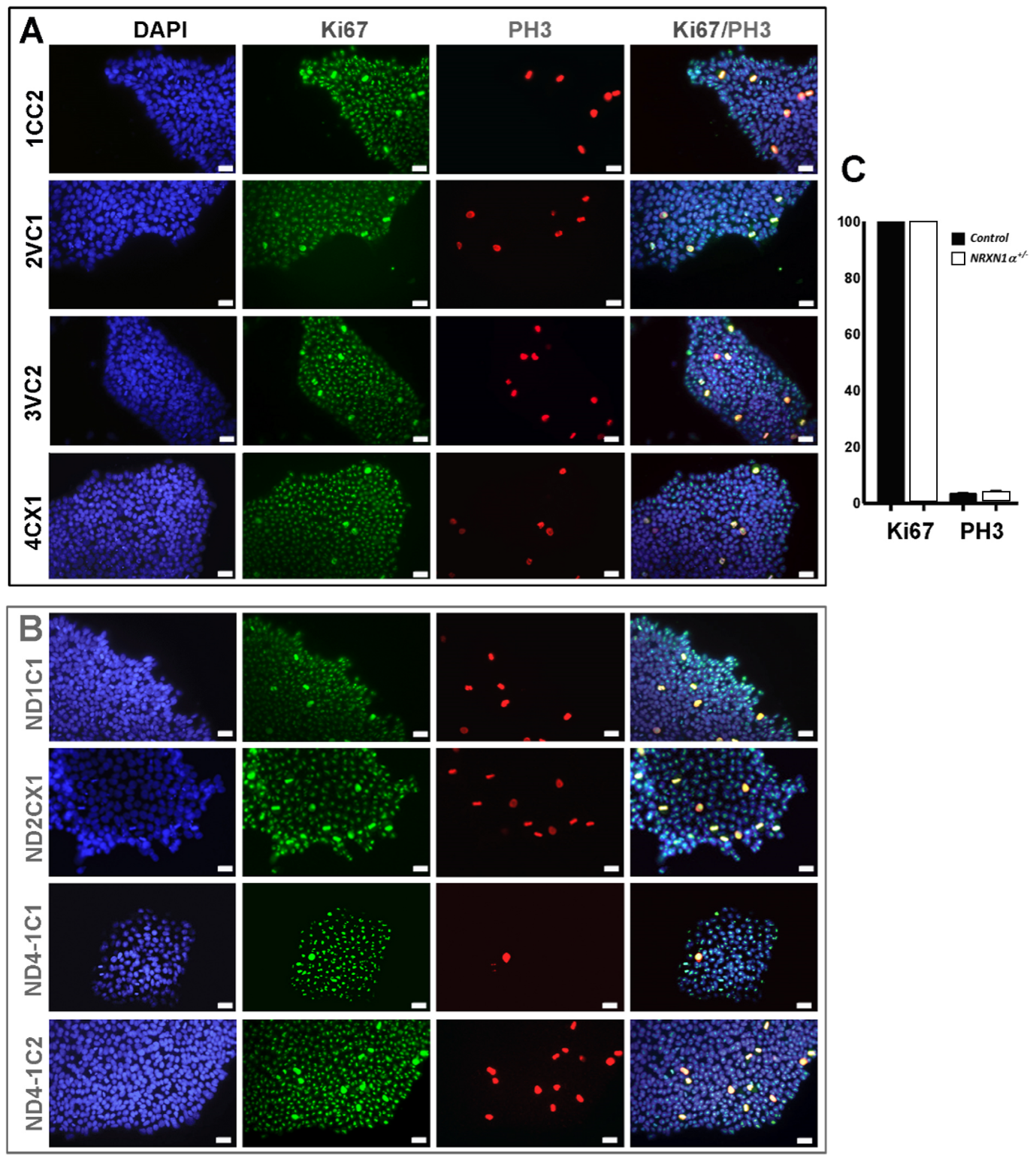
**

**Fig. S1. Proliferation of *NRXN1α^+/-^* iPSCs remained unchanged.** The iPSC from four controls (A) and four *NRXN1α^+/-^* patients (B) were stained with DAPI (blue), Ki67 (green) and PH3 (red). (C) The percentage of Ki67^+^ and PH3^+^ cells (against total DAPI) remained unchanged in *NRXN1α^+/-^* iPSCs. Scale bar 30 μm.


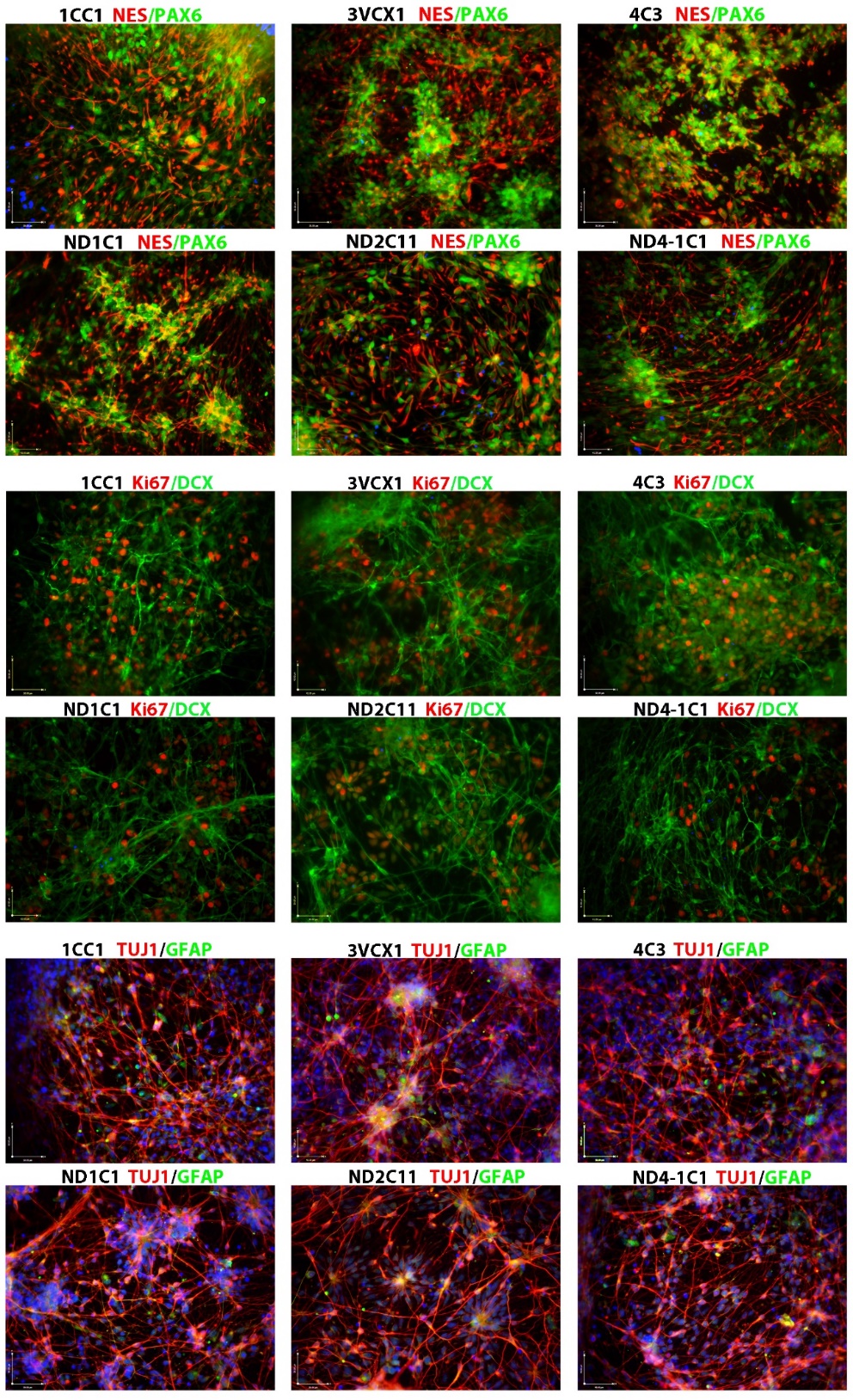


**Fig. S2. Control and *NRXN1α^+/-^* culture did not show significant difference at day 30 of neuronal differentiation.** Immunocytochemical staining was carried on day 30 cultures, with PAX6 (green) and NES (red) as neural progenitor markers, Ki67 (red) as a proliferative marker and DCX (green) for post-mitotic newborn neurons, TUJ1 (red) as pan-neuronal marker and GFAP (green) as astrocyte marker. *NRXN1α* haploinsufficiency exhibited no significant effect on proportions of PAX6***^+^***, NES***^+^***, Ki67***^+^***, DCX***^+^***, TUJ1***^+^*** or GFAP***^+^*** cells.

**Fig. S3. Functional 100 days iPSC derived neurons.** Representative images of day-100 iPSC derived-neurons stained with anti-MAP2 (A) or SYN1/TUJ1 (B) for neuronal maturity and with anti-CTIP2/TBR1 (C) for cortical identity. (D) Proportions of lower cortical glutamatergic neurons quantified from immunocytochemical images. Bars=25 µm in A-C.

**E**

| **Cell Line** | **Number of patched cells** |
| --- | --- |
| 1CC1 | 12 |
| 3VC2/3VCX1 | 14 |
| 4C3/4CX1 | 19 |
| ND1C1 | 9 |
| ND2C11/ND2 CX1 | 10 |
| ND4-1C1/ND4-1 C2 | 8 |

**Fig. S4. Voltage dependent sodium and potassium current in 100-days cultured neurons.** (A) Neurons exhibited voltage dependent sodium (A,C) and potassium (B,D) current for all controls (A,B) and *NRXN1α^+/-^* (C,D) lines. Data were shown in Mean ± SEM. The dash line separates the lines from each other. (E) The N number of patched cells for control and patient lines.

**Fig. S5.**  **Spontaneous synaptic and non-synaptic activity. (**A) Representative traces of spontaneous AP recorded at resting membrane potential confirming the excitability of cortical neurons, with zoomed in AP in B and their frequency in C (n=1-3). (D) Spontaneous EPSCs in control neurons and (E) zoomed in image of one EPSC in the blue box in the panel D. (F) A representative spontaneous EPSCs from the *NRXN1*α*^+/-^* neurons and (G) zoomed in image of one EPSC in the blue box of the panel F. Statistical analyses of the frequency (H), amplitude (I), decay time (J), rise time (K) and full width half maximum (FWHM) (L) of spontaneous EPSCs (control n=29 and *NRXN1α^+/-^* n=26). Data were shown in Mean ± SEM.

**E**

**A**


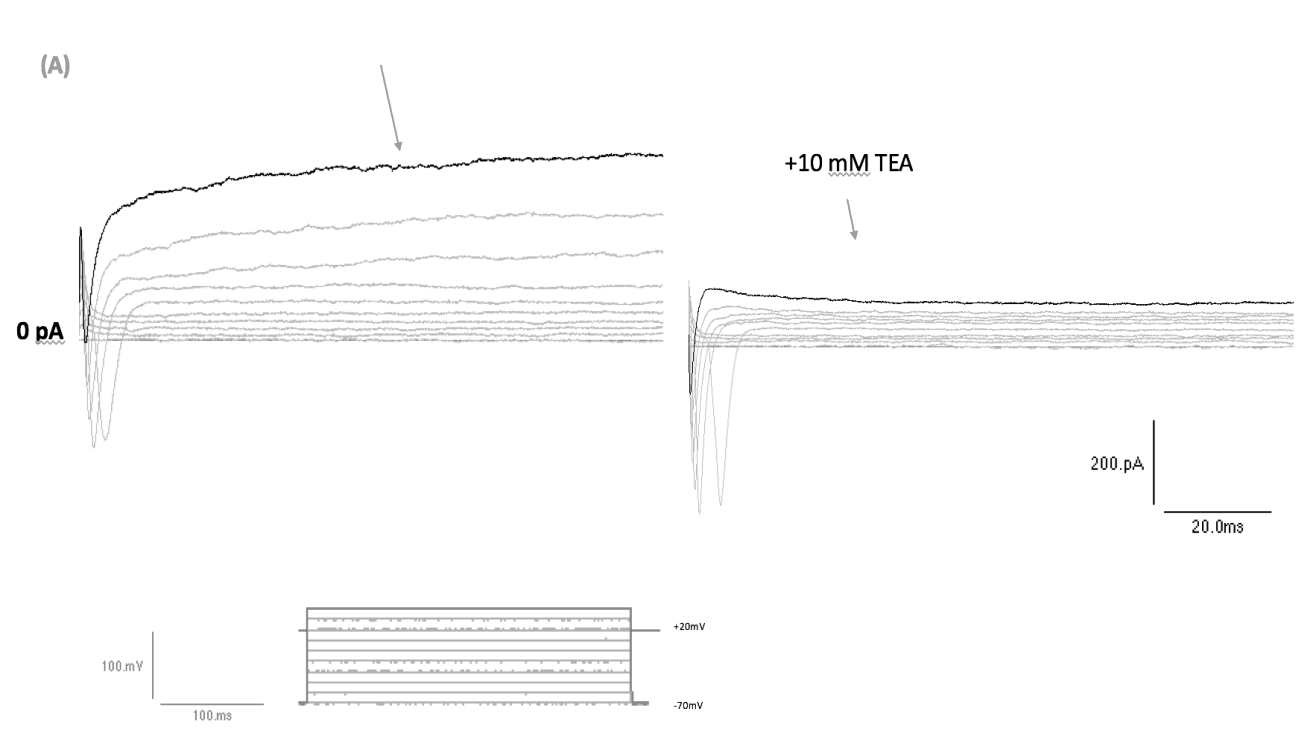


**B**


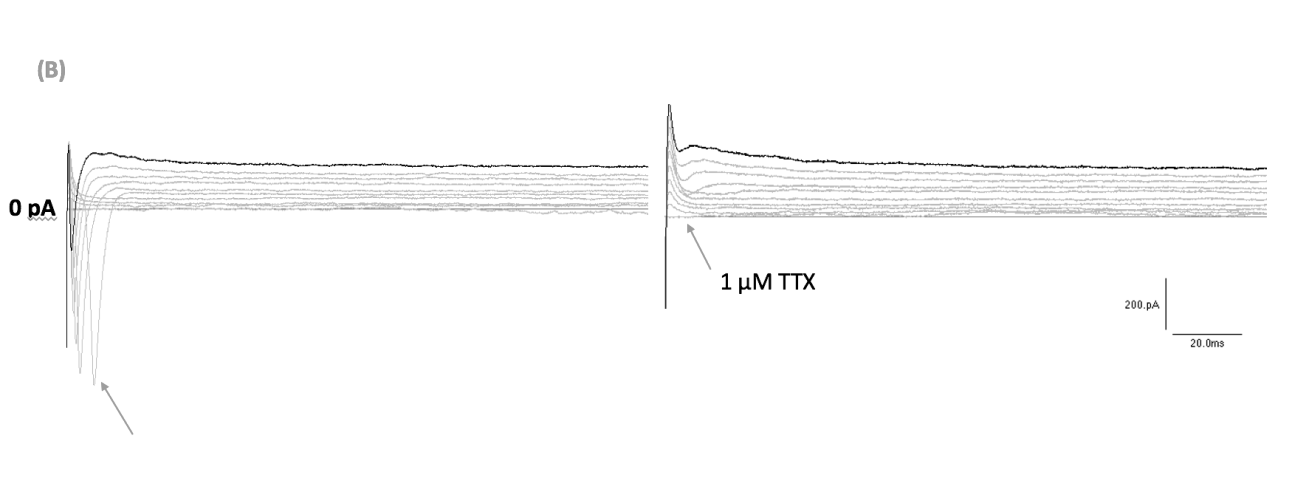


**Fig. S6. Confirmation of the identity of fast voltage activating and inactivating Na+ depolarizing currents and outward slower repolarizing K+ currents using pharmacological blockers.** Exemplar current trace, showing the application of 10 mM TEA (A) and 1µM TTX (B) with a voltage protocol shown in red.

**Table S1. List of differential expressed genes and Log_2_ changes in *NRXN1α^+/-^* neurons, with both downregulated and upregulated genes.**

| **Gene name (Downregulated)** | **log_2_ Fold Change** |
| --- | --- |
| ***NPIPA3*** | **-9.61** |
| ***HLA-DRB4*** | **-6.58** |
| ***C6*** | **-3.77** |
| ***LINC00698*** | **-3.31** |
| ***ATP6V1B1*** | **-3.27** |
| ***MSTN*** | **-2.96** |
| ***STOML3*** | **-2.94** |
| ***DTHD1*** | **-2.85** |
| ***ANKRD66*** | **-2.73** |
| ***HMCN2*** | **-2.66** |
| ***CHRDL2*** | **-2.66** |
| ***SLC47A2*** | **-2.65** |
| ***NKX6-2*** | **-2.48** |
| ***GBP2*** | **-2.44** |
| ***SRPX*** | **-2.37** |
| ***LINC01508*** | **-2.27** |
| ***PLA2G4A*** | **-2.26** |
| ***PAX8*** | **-2.25** |
| ***bNC2*** | **-2.24** |
| ***AQP4*** | **-2.23** |
| ***SLN*** | **-2.20** |
| ***CFAP73*** | **-2.19** |
| ***C2orf88*** | **-2.19** |
| ***PIRT*** | **-2.18** |
| ***LOXL4*** | **-2.13** |
| ***PROX1-AS1*** | **-2.13** |
| ***HAS2*** | **-2.12** |
| ***ITGB4*** | **-2.12** |
| ***PRRX1*** | **-2.11** |
| ***FOSL2*** | **-2.06** |
| ***GFAP*** | **-2.04** |
| ***CFAP74*** | **-2.03** |
| ***FAM166B*** | **-2.01** |
| ***CDKN2A*** | **-2.01** |
| ***PDGFRA*** | **-2.01** |
| ***CHI3L2*** | **-2.00** |
| ***CD44*** | **-2.00** |
| ***BNC2*** | **-1.95** |
| ***ADRB2*** | **-1.94** |
| ***PLSCR4*** | **-1.94** |
| ***NUPR1*** | **-1.94** |
| ***MKX*** | **-1.93** |
| ***C7orf57*** | **-1.92** |
| ***S100A10*** | **-1.92** |
| ***ROPN1L*** | **-1.90** |
| ***CXCL12*** | **-1.88** |
| ***GBP3*** | **-1.88** |
| ***GRHL3*** | **-1.86** |
| ***DNAH9*** | **-1.85** |
| ***OSTM1-AS1*** | **-1.84** |
| ***SMOC2*** | **-1.82** |
| ***DAW1*** | **-1.82** |
| ***EFHD1*** | **-1.78** |
| ***CFAP161*** | **-1.77** |
| ***SPAG6*** | **-1.76** |
| ***SLC34A2*** | **-1.76** |
| ***FAM129A*** | **-1.76** |
| ***KIAA1211L*** | **-1.76** |
| ***CFAP52*** | **-1.76** |
| ***FAM183A*** | **-1.75** |
| ***TRIM47*** | **-1.74** |
| ***CERKL*** | **-1.74** |
| ***APOBEC3B*** | **-1.73** |
| ***PODN*** | **-1.73** |
| ***FAM47E-STBD1*** | **-1.71** |
| ***TCTEX1D1*** | **-1.71** |
| ***SYTL2*** | **-1.70** |
| ***TTC29*** | **-1.70** |
| ***CRYAB*** | **-1.69** |
| ***MMP19*** | **-1.69** |
| ***CFB*** | **-1.68** |
| ***AGT*** | **-1.67** |
| ***LRRC71*** | **-1.67** |
| ***GALNT15*** | **-1.67** |
| ***TNC*** | **-1.66** |
| ***TPPP3*** | **-1.66** |
| ***SMIM5*** | **-1.65** |
| ***CLEC3B*** | **-1.65** |
| ***GBP1*** | **-1.65** |
| ***MAP3K19*** | **-1.64** |
| ***WDR38*** | **-1.64** |
| ***S1PR3*** | **-1.64** |
| ***RUNX1*** | **-1.62** |
| ***FAM81B*** | **-1.61** |
| ***NTN1*** | **-1.60** |
| ***PRSS35*** | **-1.59** |
| ***HKDC1*** | **-1.58** |
| ***CAV2*** | **-1.58** |
| ***CFAP77*** | **-1.57** |
| ***MORN5*** | **-1.57** |
| ***DRC1*** | **-1.54** |
| ***SPTSSB*** | **-1.54** |
| ***SCRG1*** | **-1.53** |
| ***CAV1*** | **-1.53** |
| ***RSPH1*** | **-1.53** |
| ***TGFBR3*** | **-1.53** |
| ***GJA1*** | **-1.52** |
| ***CPZ*** | **-1.52** |
| ***C16orf89*** | **-1.50** |
| ***TEKT1*** | **-1.50** |
| ***OSMR*** | **-1.50** |
| ***ANXA1*** | **-1.49** |
| ***CEBPD*** | **-1.49** |
| ***EFHB*** | **-1.49** |
| ***VWA3B*** | **-1.49** |
| ***MFAP4*** | **-1.48** |
| ***HRH1*** | **-1.48** |
| ***C11orf88*** | **-1.47** |
| ***CLEC19A*** | **-1.46** |
| ***JAKMIP2-AS1*** | **-1.46** |
| ***RARRES3*** | **-1.45** |
| ***CHST9*** | **-1.45** |
| ***NFIA-AS2*** | **-1.45** |
| ***LOC100506100*** | **-1.44** |
| ***ANGPT1*** | **-1.44** |
| ***ZMYND10*** | **-1.43** |
| ***ABI3BP*** | **-1.42** |
| ***MNS1*** | **-1.42** |
| ***LRRC46*** | **-1.41** |
| ***FAM216B*** | **-1.41** |
| ***ADAMTS16*** | **-1.41** |
| ***DYNLRB2*** | **-1.40** |
| ***ST6GALNAC2*** | **-1.40** |
| ***STEAP3*** | **-1.40** |
| ***LRRN4CL*** | **-1.40** |
| ***FBXO32*** | **-1.40** |
| ***TGFB2*** | **-1.40** |
| ***PDLIM1*** | **-1.40** |
| ***AK7*** | **-1.39** |
| ***FANK1*** | **-1.39** |
| ***SLC35D2*** | **-1.39** |
| ***HTR1D*** | **-1.37** |
| ***CD38*** | **-1.36** |
| ***LINC00880*** | **-1.36** |
| ***CLU*** | **-1.36** |
| ***KCNJ8*** | **-1.36** |
| ***TSPAN12*** | **-1.35** |
| ***DAB2*** | **-1.35** |
| ***PRR29*** | **-1.35** |
| ***KCNJ10*** | **-1.33** |
| ***ELL2*** | **-1.32** |
| ***APLN*** | **-1.32** |
| ***GADD45B*** | **-1.32** |
| ***SSPN*** | **-1.31** |
| ***CFAP54*** | **-1.31** |
| ***EPAS1*** | **-1.31** |
| ***CFAP45*** | **-1.30** |
| ***GCNT4*** | **-1.30** |
| ***F2RL2*** | **-1.30** |
| ***PMP22*** | **-1.29** |
| ***FAM134B*** | **-1.26** |
| ***ARSJ*** | **-1.26** |
| ***NFATC1*** | **-1.26** |
| ***ZFP36*** | **-1.26** |
| ***RBM20*** | **-1.25** |
| ***P3H2*** | **-1.25** |
| ***FOXJ1*** | **-1.25** |
| ***SNTB1*** | **-1.25** |
| ***C5orf49*** | **-1.25** |
| ***CYYR1*** | **-1.25** |
| ***ARMC3*** | **-1.24** |
| ***SYNPO*** | **-1.24** |
| ***ANXA5*** | **-1.24** |
| ***LIMS2*** | **-1.22** |
| ***CST3*** | **-1.22** |
| ***PLSCR1*** | **-1.21** |
| ***LINC01354*** | **-1.21** |
| ***ITPR2*** | **-1.20** |
| ***SPATA18*** | **-1.19** |
| ***ZNF474*** | **-1.19** |
| ***CYBRD1*** | **-1.19** |
| ***PRR18*** | **-1.19** |
| ***GABRE*** | **-1.19** |
| ***SPATA17*** | **-1.19** |
| ***ITPKB*** | **-1.18** |
| ***PIFO*** | **-1.18** |
| ***LRRIQ1*** | **-1.18** |
| ***GYPC*** | **-1.18** |
| ***MAN1A1*** | **-1.17** |
| ***RASSF9*** | **-1.17** |
| ***C10orf107*** | **-1.17** |
| ***IL1RAP*** | **-1.16** |
| ***PROX1*** | **-1.16** |
| ***SLC26A7*** | **-1.16** |
| ***MOXD1*** | **-1.16** |
| ***DYX1C1-CCPG1*** | **-1.16** |
| ***NFIX*** | **-1.16** |
| ***RIN3*** | **-1.16** |
| ***GLIS3*** | **-1.15** |
| ***AAED1*** | **-1.14** |
| ***MRO*** | **-1.14** |
| ***CCDC102A*** | **-1.14** |
| ***CDKN1A*** | **-1.14** |
| ***CD55*** | **-1.13** |
| ***PDGFA*** | **-1.13** |
| ***ATF3*** | **-1.13** |
| ***ODF3B*** | **-1.13** |
| ***GPX3*** | **-1.13** |
| ***PDPN*** | **-1.13** |
| ***TFEB*** | **-1.13** |
| ***SYNC*** | **-1.12** |
| ***FAS*** | **-1.12** |
| ***TNFSF12*** | **-1.12** |
| ***SNX7*** | **-1.12** |
| ***CFAP206*** | **-1.12** |
| ***LRRC9*** | **-1.11** |
| ***SULT1C4*** | **-1.11** |
| ***LAMP3*** | **-1.11** |
| ***HERC5*** | **-1.11** |
| ***GMPR*** | **-1.11** |
| ***GXYLT2*** | **-1.11** |
| ***PLPP1*** | **-1.10** |
| ***FBLN7*** | **-1.10** |
| ***NEXN*** | **-1.10** |
| ***CTSO*** | **-1.10** |
| ***TGFBR2*** | **-1.10** |
| ***FCGRT*** | **-1.10** |
| ***PROS1*** | **-1.10** |
| ***EVA1C*** | **-1.09** |
| ***CTF1*** | **-1.09** |
| ***GPR132*** | **-1.09** |
| ***WDR63*** | **-1.09** |
| ***DRC7*** | **-1.08** |
| ***C21orf62*** | **-1.08** |
| ***GRIN3B*** | **-1.08** |
| ***PON2*** | **-1.08** |
| ***TRIB3*** | **-1.08** |
| ***ITM2C*** | **-1.07** |
| ***ME1*** | **-1.07** |
| ***CFAP126*** | **-1.07** |
| ***CRISPLD1*** | **-1.07** |
| ***CD58*** | **-1.06** |
| ***NRP1*** | **-1.06** |
| ***PARP12*** | **-1.06** |
| ***TGFB1*** | **-1.06** |
| ***PARM1*** | **-1.05** |
| ***HLA-E*** | **-1.05** |
| ***LOX*** | **-1.05** |
| ***ADAMTS15*** | **-1.05** |
| ***ZC3H12A*** | **-1.05** |
| ***OLFML2A*** | **-1.05** |
| ***PRSS23*** | **-1.04** |
| ***CD99*** | **-1.04** |
| ***CEBPB*** | **-1.04** |
| ***ARHGAP29*** | **-1.04** |
| ***SPAG1*** | **-1.04** |
| ***GALNT18*** | **-1.04** |
| ***C1orf198*** | **-1.03** |
| ***CASC1*** | **-1.03** |
| ***NEBL*** | **-1.03** |
| ***AHNAK2*** | **-1.02** |
| ***FILIP1L*** | **-1.02** |
| ***TMEM35B*** | **-1.02** |
| ***SERPING1*** | **-1.02** |
| ***RPGR*** | **-1.02** |
| ***EMP3*** | **-1.01** |
| ***PLEKHA2*** | **-1.01** |
| ***TMBIM1*** | **-1.01** |
| ***TMEM47*** | **-1.01** |
| ***GEM*** | **-1.01** |
| ***BVES*** | **-1.01** |
| ***CD9*** | **-1.00** |
| ***GADD45A*** | **-1.00** |
| ***NEK6*** | **-1.00** |
| ***SCARA3*** | **-1.00** |
| ***CAPS*** | **-1.00** |
| ***NID1*** | **-1.00** |
| ***BMPR1B*** | **-1.00** |
| ***PLCD3*** | **-1.00** |
| **Gene name (Upregulated)** | **log_2_ Fold Change** |
| ***EPHA10*** | 1.00 |
| ***CNTN2*** | 1.00 |
| ***COL7A1*** | 1.00 |
| ***CD8A*** | 1.00 |
| ***PPP1R16B*** | 1.00 |
| ***GUSBP9*** | 1.00 |
| ***KRT8*** | 1.01 |
| ***ELAVL2*** | 1.01 |
| ***SYT3*** | 1.01 |
| ***CACNA1A*** | 1.01 |
| ***CACNA1I*** | 1.02 |
| ***CKMT1B*** | 1.02 |
| ***BICDL1*** | 1.02 |
| ***GRIP2*** | 1.02 |
| ***WNT7A*** | 1.02 |
| ***SHISA7*** | 1.02 |
| ***TRIM7*** | 1.03 |
| ***RAB3C*** | 1.03 |
| ***SHANK1*** | 1.03 |
| ***NECAB2*** | 1.03 |
| ***AMER3*** | 1.03 |
| ***SLC8A3*** | 1.03 |
| ***KCNB2*** | 1.03 |
| ***CPNE5*** | 1.04 |
| ***PTCH2*** | 1.04 |
| ***NPTN-IT1*** | 1.05 |
| ***CDH7*** | 1.06 |
| ***FOXH1*** | 1.07 |
| ***KCNF1*** | 1.07 |
| ***RAPGEF5*** | 1.08 |
| ***CACNA2D2*** | 1.08 |
| ***CNTNAP2*** | 1.08 |
| ***RYR1*** | 1.09 |
| ***CCDC88B*** | 1.09 |
| ***CACNG2*** | 1.09 |
| ***PTPN5*** | 1.10 |
| ***LINC00176*** | 1.10 |
| ***SLC6A17*** | 1.11 |
| ***MANEA-AS1*** | 1.12 |
| ***SYT13*** | 1.12 |
| ***TMEM266*** | 1.12 |
| ***BMP8A*** | 1.12 |
| ***KCNK12*** | 1.13 |
| ***HPCAL4*** | 1.14 |
| ***LMX1A*** | 1.14 |
| ***SYNJ2*** | 1.14 |
| ***CADM3-AS1*** | 1.15 |
| ***KCNK6*** | 1.15 |
| ***FUT1*** | 1.15 |
| ***OGDHL*** | 1.16 |
| ***FGF17*** | 1.16 |
| ***LINC00599*** | 1.16 |
| ***CDH12*** | 1.16 |
| ***DRP2*** | 1.16 |
| ***SLC7A4*** | 1.17 |
| ***ZNF804A*** | 1.17 |
| ***SIAH3*** | 1.18 |
| ***ABLIM3*** | 1.19 |
| ***CCDC85A*** | 1.19 |
| ***FAM19A2*** | 1.20 |
| ***SLC7A14*** | 1.20 |
| ***LINC01783*** | 1.21 |
| ***RASGRF1*** | 1.22 |
| ***CUX2*** | 1.22 |
| ***KCNK9*** | 1.23 |
| ***STXBP5-AS1*** | 1.23 |
| ***ADARB2*** | 1.23 |
| ***DDN*** | 1.24 |
| ***TRH*** | 1.24 |
| ***GUCY1A3*** | 1.24 |
| ***HAS3*** | 1.24 |
| ***CHST8*** | 1.24 |
| ***THCAT155*** | 1.24 |
| ***C17orf107*** | 1.25 |
| ***ANK1*** | 1.25 |
| ***GRM1*** | 1.25 |
| ***CDH18*** | 1.26 |
| ***VWA5B2*** | 1.27 |
| ***TMEM145*** | 1.28 |
| ***KCNMB2*** | 1.28 |
| ***CLMP*** | 1.30 |
| ***OLFM3*** | 1.30 |
| ***SOWAHA*** | 1.31 |
| ***AFDN-AS1*** | 1.32 |
| ***CSMD1*** | 1.33 |
| ***WSCD2*** | 1.33 |
| ***NDST3*** | 1.33 |
| ***SYN3*** | 1.34 |
| ***DCTN1-AS1*** | 1.34 |
| ***DCLK3*** | 1.36 |
| ***PCDH11Y*** | 1.37 |
| ***HAR1A*** | 1.38 |
| ***KIAA1024*** | 1.38 |
| ***GOLGA6L3*** | 1.39 |
| ***LGI2*** | 1.39 |
| ***NEGR1*** | 1.39 |
| ***OMG*** | 1.40 |
| ***HPX*** | 1.41 |
| ***NMU*** | 1.41 |
| ***ZDHHC8P1*** | 1.42 |
| ***CHD5*** | 1.43 |
| ***CABP7*** | 1.43 |
| ***ACTG1P4*** | 1.46 |
| ***CYBA*** | 1.46 |
| ***EPHA8*** | 1.47 |
| ***POU3F1*** | 1.48 |
| ***TRPM3*** | 1.49 |
| ***LMO3*** | 1.50 |
| ***TMEM179*** | 1.51 |
| ***WNT3A*** | 1.51 |
| ***KCNJ5*** | 1.53 |
| ***CDH22*** | 1.56 |
| ***HS3ST5*** | 1.56 |
| ***RSPO1*** | 1.57 |
| ***PCDH11X*** | 1.58 |
| ***TRIM58*** | 1.59 |
| ***CPNE7*** | 1.61 |
| ***PPIEL*** | 1.63 |
| ***MIR124-2HG*** | 1.71 |
| ***CACNG3*** | 1.72 |
| ***LPL*** | 1.72 |
| ***CNTNAP5*** | 1.72 |
| ***HS3ST2*** | 1.74 |
| ***HPGD*** | 1.75 |
| ***BTNL9*** | 1.78 |
| ***FSTL5*** | 1.84 |
| ***GPR26*** | 1.85 |
| ***SLC17A6*** | 1.85 |
| ***SLC12A5*** | 1.86 |
| ***PCDHGC4*** | 1.86 |
| ***GDF7*** | 1.88 |
| ***SSTR1*** | 1.90 |
| ***TSTD1*** | 1.98 |
| ***KLHL1*** | 1.99 |
| ***CNTNAP4*** | 2.02 |
| ***MAL*** | 2.04 |
| ***GPR179*** | 2.05 |
| ***GABRA1*** | 2.06 |
| ***TH*** | 2.08 |
| ***PTER*** | 2.09 |
| ***CHRM2*** | 2.12 |
| ***RTP5*** | 2.15 |
| ***SCG5*** | 2.15 |
| ***LOC646241*** | 2.16 |
| ***SNCG*** | 2.23 |
| ***SPOCK3*** | 2.24 |
| ***PDYN*** | 2.34 |
| ***SHISA8*** | 2.38 |
| ***C1QL3*** | 2.58 |
| ***GJD2*** | 2.88 |
| ***PMCH*** | 3.11 |
| ***AKAIN1*** | 3.31 |
| ***CPNE6*** | 3.47 |
| ***CARTPT*** | 3.68 |
| ***HCRTR2*** | 3.86 |
| ***FAM226A*** | 7.18 |
